# Crystal structure of bis­[(*R*,*R*)-1,2-(bi­naph­thyl­phospho­nito)ethane]­dichlorido­iron(II) di­chloro­methane disolvate

**DOI:** 10.1107/S2056989020011160

**Published:** 2020-08-28

**Authors:** Benjamin E. Rennie, Alan J. Lough, Robert H. Morris

**Affiliations:** a Department of Chemistry, University of Toronto, Toronto, Ontario, M5S 3H6, Canada

**Keywords:** crystal structure, BINAP, BINOL, asymmetric catalysis

## Abstract

In the title compound (systematic name: bis­{1,2-bis[12,14-dioxa-13-phospha­penta­cyclo­[13.8.0.0^2,11^.0^3,8^.0^18,23^]tricosa-1(15),2(11),3(8),4,6,9,16,18(23),19,21-deca­en-13-yl]ethane}­dichlorido­iron(II) di­chloro­methane disolvate), [FeCl_2_(C_42_H_28_O_4_P_2_)_2_]·2CH_2_Cl_2_, the Fe^II^ ion lies on a crystallographic twofold rotation axis and is coordinated by four P atoms from two (*R,R*)-1,2-bis­(bi­naphthyl­phospho­n­ito)ethane (BPE) ligands and two Cl ligands in a distorted *cis*-FeCl_2_P_4_ octa­hedral coordination geometry. In the crystal, weak C—H⋯O and C—H⋯π inter­actions link the mol­ecules into layers lying parallel to (001). A weak intra­molecular C—H⋯O hydrogen bond is also observed. The asymmetric unit contains one CH_2_Cl_2_ solvent mol­ecule, which is disordered over two sets of site with refined occupancies in the ratio 0.700 (6):0.300 (6).

## Abstract 2

In the title compound, the Fe^II^ ion lies on a crystallographic twofold rotation axis and is coordinated by four P atoms from two (*R*,*R*)-1,2-bis­(bi­naphthyl­phospho­nito)ethane (BPE) ligands and two Cl ligands in a distorted *cis*-FeCl_2_P_4_ octa­hedral coordination geometry. Weak C—H⋯O and C—H⋯π inter­actions occur in the crystal.

## Chemical context   

The ligand (*R*,*R*)- or (*S*,*S*)-1,2-bis­(bi­naphthyl­phospho­nito)ethane (C_42_H_28_O_4_P_2_; BPE) prepared from either (*R*)- or (*S*)-1,1′-bi(2-naphthol) (C_20_H_14_O_2_; BINOL) has been used extensively in asymmetric catalysis, as has the related ligand (*R*) or (*S*)-2,2′-bis­(di­phenyl­phosphino)-1,1′-binaphthyl (C_44_H_32_P_2_; BINAP). For example, the BINAP ligand has been coordinated to ruthenium and used for the asymmetric hydrogenation of ketones (Doucet *et al.*, 1998 ▸), among many other examples. The BINAP ligand has also been coordinated to iron (Vogler, 2016 ▸) to make the complex [FeCl_2_(BINAP)_2_]. The BPE ligand and similar bidentate and monodentate phospho­nite ligands have been coordinated to rhodium and iridium and used for asymmetric alkene and quinoline hydrogenation reactions, respectively (Claver *et al.*, 2000 ▸; Norman *et al.*, 2008 ▸; Reetz & Li, 2006 ▸), and to ruthenium for asymmetric transfer hydrogenation (Guo *et al.*, 2005*a* ▸,*b* ▸).
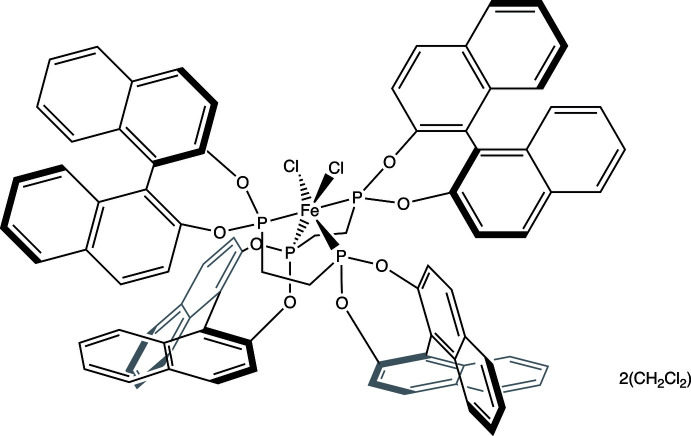


As an extension of these studies, we now describe the synthesis and crystal structure of the iron(II) complex FeCl_2_(BPE)_2_, which crystallized as a di­chloro­methane solvate.

## Structural commentary   

**Figure 1:**
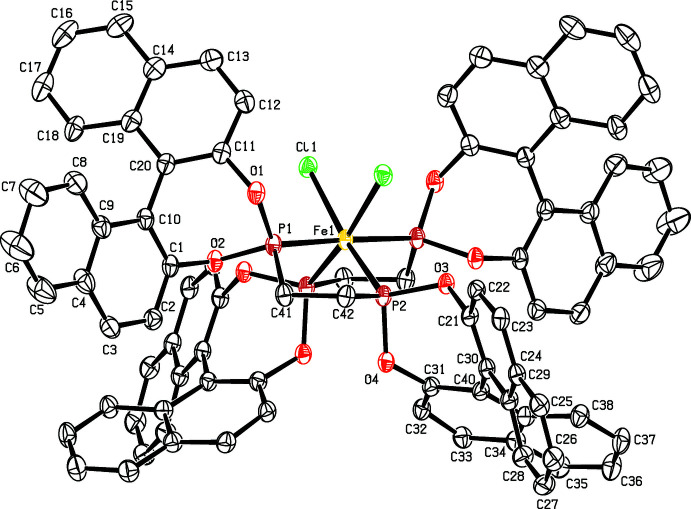
The mol­ecular structure of the title compound with 30% probability ellipsoids. Unlabeled atoms are related by the symmetry operator (*y*, *x*, −*z* + 1) and for the sake of clarity the disordered solvent mol­ecule is not shown.

The mol­ecular structure of the title compound is shown in Fig. 1 ▸. The Fe^II^ ion lies on a crystallographic twofold rotation axis and is coordinated by four P atoms from two BPE ligands and two Cl ligands in a distorted *cis*-FeCl_2_P_4_ octa­hedral coordination geometry. The largest distortion from ideal coordination geometry is the P2—Fe—P2^i^ angle of 108.49 (7)° (see Table 1 ▸ for symmetry codes). The distortion is based on steric grounds involving the bulky bi­naphthyl­phospho­nito ligands. The Fe—P distances are the same within experimental error. The P atoms are bonded to two O atoms, one C atom and coordinated to the Fe^II^ ion in distorted tetra­hedral geometries. The dihedral angles between the naphthalene rings in the BPE ligands (C1–C10/C11–20 and C21–C30/C31–C40) are the same, with values of 54.5 (2)°. A weak intra­molecular C—H⋯O hydrogen bond is observed (Table 2 ▸). The asymmetric unit contains one CH_2_Cl_2_ solvent mol­ecule, which is disordered over two sets of sites with refined occupancies in the ratio 0.700 (6):0.300 (6).

## Supra­molecular features   

**Figure 2:**
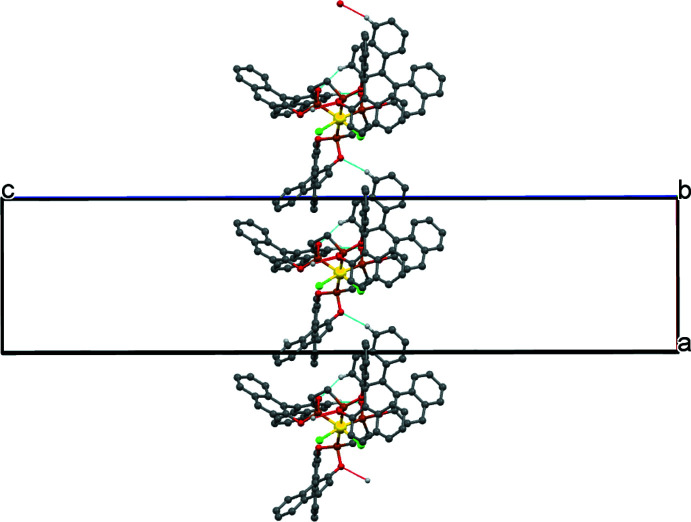
Part of the crystal structure of the title compound showing the formation of [100] chains linked by weak C—H⋯O hydrogen bonds shown as blue lines. The disordered di­chloro­methane solvent mol­ecules are not shown.

In the crystal, weak C—H⋯O hydrogen bonds link mol­ecules into sheets parallel to (001) (Table 2 ▸ and Fig. 2 ▸). Within these layers weak C—H⋯π inter­actions also occur, and the centroid–centroid distance *Cg*2⋯*Cg*2(*y*, −1 + *x*, 1 − *z*) of 4.171 (5) Å (where *Cg*2 is the centroid of the C4–C9 benzene ring) may be a very weak π-stacking inter­action.

## Database survey   

A search of the Cambridge Structural Database (CSD, Version 5.41, November, 2019; Groom *et al.*, 2016 ▸) showed surprisingly that the title complex is the first iron(II) dichloride crystal structure with bidentate phospho­rus donors with P—O-bonded substituents. There are 36 structures of related iron diphosphine complexes FeCl_2_(P_2_)_2_ (P_2_ = a diphosphine) with P—C bonds reported. The majority, 33 complexes, crystallize with the chloride ions *trans* to each other, while there are three examples where the chloride ions are *cis*, as in the title complex. The complex *trans*-FeCl_2_(1,2-bis­(di­phenyl­phosphino)ethyl­ene)_2_, for example, crystallizes with the chloride ions *trans* (Cecconi *et al.*, 1981 ▸). An example with *cis* chloride ions is the complex *cis*-FeCl_2_(1,2-di­phospho­lano­ethane)_2_ (Field *et al.*, 1998 ▸). In the *trans* complexes, the Fe—Cl distances range from 2.21 to 2.38 Å with 22 structures having a distance of 2.34–2.37 Å. This compares with the distances of 2.3422 (11) and 2.3423 (11) Å in the title complex.

## Synthesis and crystallization   

The ligand was synthesized according to a literature procedure using (*R*)-BINOL (Steinmetz *et al.*, 1999 ▸). The iron complex was synthesized as follows: in a nitro­gen-filled glovebox, FeCl_2_·1.5THF (6.0 mg, 0.030 mmol, 1 equivalent) was combined with *(R*,*R*)-BPE (50 mg, 0.08 mmol, 3 equivalents) in 10 ml THF and stirred in a 20 ml dram vial for 24 h. The THF was vacuumed off to yield a brown powder: ^31^P{^1^H} NMR (202 MHz, C_6_D_6_): 257.72 ppm, singlet.

To purify, the powder was dissolved in a minimum of DCM, precipitated out with addition of diethyl ether, and filtered over a glass frit. The precipitate collected on the frit was re-dissolved in DCM, and re-purified by the same procedure twice more. To obtain crystals, a concentrated DCM solution of the purified complex was left in a closed 20 ml dram vial in a nitro­gen-filled glovebox for approximately one week at least, depending on the exact concentration. The crystals were orange coloured. Attempts to convert this complex into a hydride complex were unsuccessful.

## Refinement   

Crystal data, data collection and structure refinement details are summarized in Table 3 ▸. H atoms were included in calculated positions with C—H = 0.95 and 0.99 Å for aromatic and methyl­ene C atoms, respectively, and were included in a riding-model approximation with *U*_iso_(H) = 1.2*U*_eq_(C).

The major component of the disordered CH_2_Cl_2_ solvent mol­ecule was refined without restraints while the minor component was restrained to have similar geometry and anisotropic displacement parameters to the major component using the SAME and SADI instructions in *SHELXL* (Sheldrick, 2015*b* ▸).

## Supplementary Material

Crystal structure: contains datablock(s) I. DOI: 10.1107/S2056989020011160/hb7939sup1.cif

Structure factors: contains datablock(s) I. DOI: 10.1107/S2056989020011160/hb7939Isup2.hkl

CCDC reference: 2023248

Additional supporting information:  crystallographic information; 3D view; checkCIF report

## Figures and Tables

**Table 1:** Selected geometric parameters (Å, °)

Fe1—P2	2.1594 (11)	Fe1—P1	2.1952 (10)
Fe1—P2^i^	2.1595 (11)	Fe1—Cl1^i^	2.3422 (11)
Fe1—P1^i^	2.1952 (10)	Fe1—Cl1	2.3423 (11)
			
P2—Fe1—P2^i^	108.49 (7)	P1—Fe1—Cl1^i^	88.52 (4)
P2—Fe1—P1^i^	93.40 (4)	P2—Fe1—Cl1	170.02 (5)
P2—Fe1—P1	85.30 (4)	P1—Fe1—Cl1	93.07 (4)
P1^i^—Fe1—P1	177.78 (7)	Cl1^i^—Fe1—Cl1	88.69 (6)
P2—Fe1—Cl1^i^	81.43 (4)		

Symmetry code: (i) 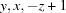.

**Table 2:** Hydrogen-bond geometry (Å, °) *Cg*2 and *Cg*3 are the centroids of the C24–C29 and C31–C40 rings, respectively.

*D*—H⋯*A*	*D*—H	H⋯*A*	*D*⋯*A*	*D*—H⋯*A*
C32—H32*A*⋯O4^i^	0.95	2.42	3.280 (5)	150
C35—H35*A*⋯O1^ii^	0.95	2.38	3.293 (5)	162
C7—H7*A*⋯*Cg*2^iii^	0.95	2.57	3.516 (6)	178
C17—H17*A*⋯*Cg*3^iii^	0.95	2.59	3.396 (6)	143

Symmetry codes: (i) 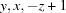; (ii) 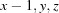; (iii) 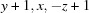.

**Table 3:** Experimental details

Crystal data	
Chemical formula	[FeCl_2_(C_42_H_28_O_4_P_2_)_2_]·2CH_2_Cl_2_
*M*_r_	1613.77
Crystal system, space group	Tetragonal, *P*4_3_2_1_2
Temperature (K)	150
*a*, *c* (Å)	11.9850 (3), 52.4508 (14)
*V* (Å^3^)	7534.0 (4)
*Z*	4
Radiation type	Cu *K*α
μ (mm^−1^)	4.84
Crystal size (mm)	0.09 × 0.04 × 0.02

Data collection	
Diffractometer	Bruker Kappa *APEX* DUO CCD
Absorption correction	Multi-scan (*SADABS*; Krause *et al.*, 2015 ▸)
*T*_min_, *T*_max_	0.649, 0.740
No. of measured, independent and observed [*I* > 2σ(*I*)] reflections	97444, 6829, 6096
*R*_int_	0.109
(sin θ/λ)_max_ (Å^−1^)	0.600

Refinement	
*R*[*F* ^2^ > 2σ(*F* ^2^)], *wR*(*F* ^2^), *S*	0.043, 0.110, 1.04
No. of reflections	6829
No. of parameters	502
No. of restraints	51
H-atom treatment	H-atom parameters constrained
Δρ_max_, Δρ_min_ (e Å^−3^)	0.39, −0.65
Absolute structure	Flack *x* determined using 2237 quotients [(*I* ^+^)−(*I* ^−^)]/[(*I* ^+^)+(*I* ^−^)] (Parsons *et al.*, 2013 ▸)
Absolute structure parameter	0.004 (4)

Computer programs: *APEX3* and *SAINT* (Bruker, 2018 ▸), *SHELXT2014/5* (Sheldrick, 2015*a* ▸), *SHELXL2018/3* (Sheldrick, 2015*b* ▸), *PLATON* (Spek, 2020 ▸) and *SHELXTL* (Sheldrick, 2008 ▸).

## supplementary crystallographic information

### Crystal data

**Table:**

[FeCl_2_(C_42_H_28_O_4_P_2_)_2_]·2CH_2_Cl_2_	*D*_x_ = 1.423 Mg m^−^^3^
*M**_r_* = 1613.77	Cu *K*α radiation, λ = 1.54178 Å
Tetragonal, *P*4_3_2_1_2	Cell parameters from 6128 reflections
*a* = 11.9850 (3) Å	θ = 3.4–67.3°
*c* = 52.4508 (14) Å	µ = 4.84 mm^−^^1^
*V* = 7534.0 (4) Å^3^	*T* = 150 K
*Z* = 4	Shard, orange
*F*(000) = 3312	0.09 × 0.04 × 0.02 mm

### Data collection

**Table:**

Bruker Kappa APEX DUO CCD diffractometer	6096 reflections with *I* > 2σ(*I*)
Radiation source: Bruker ImuS with multi-layer optics	*R*_int_ = 0.109
φ and ω scans	θ_max_ = 67.8°, θ_min_ = 3.4°
Absorption correction: multi-scan (SADABS; Krause *et al.*, 2015)	*h* = −14→14
*T*_min_ = 0.649, *T*_max_ = 0.740	*k* = −14→14
97444 measured reflections	*l* = −62→60
6829 independent reflections	

### Refinement

**Table:**

Refinement on *F*^2^	Hydrogen site location: inferred from neighbouring sites
Least-squares matrix: full	H-atom parameters constrained
*R*[*F*^2^ > 2σ(*F*^2^)] = 0.043	*w* = 1/[σ^2^(*F*_o_^2^) + (0.0538*P*)^2^ + 2.6304*P*] where *P* = (*F*_o_^2^ + 2*F*_c_^2^)/3
*wR*(*F*^2^) = 0.110	(Δ/σ)_max_ = 0.002
*S* = 1.04	Δρ_max_ = 0.39 e Å^−^^3^
6829 reflections	Δρ_min_ = −0.65 e Å^−^^3^
502 parameters	Absolute structure: Flack *x* determined using 2237 quotients [(*I*^+^)-(*I*^-^)]/[(*I*^+^)+(*I*^-^)] (Parsons *et al.*, 2013)
51 restraints	Absolute structure parameter: 0.004 (4)
Primary atom site location: structure-invariant direct methods	

### Special details

**Table:**

Geometry. All esds (except the esd in the dihedral angle between two l.s. planes) are estimated using the full covariance matrix. The cell esds are taken into account individually in the estimation of esds in distances, angles and torsion angles; correlations between esds in cell parameters are only used when they are defined by crystal symmetry. An approximate (isotropic) treatment of cell esds is used for estimating esds involving l.s. planes.

### Fractional atomic coordinates and isotropic or equivalent isotropic displacement parameters (Å^2^)

**Table:**

	*x*	*y*	*z*	*U*_iso_*/*U*_eq_	Occ. (<1)
Fe1	0.48561 (5)	0.48561 (5)	0.500000	0.0307 (2)	
Cl1	0.56836 (9)	0.60053 (9)	0.53078 (2)	0.0399 (2)	
P1	0.61181 (9)	0.35440 (9)	0.50461 (2)	0.0337 (2)	
P2	0.43146 (9)	0.39087 (9)	0.46724 (2)	0.0323 (2)	
O1	0.7416 (2)	0.3808 (3)	0.49888 (5)	0.0372 (6)	
O2	0.6217 (2)	0.2921 (2)	0.53178 (5)	0.0353 (6)	
O3	0.4019 (2)	0.4596 (2)	0.44174 (5)	0.0339 (6)	
O4	0.3269 (2)	0.3037 (2)	0.46814 (5)	0.0344 (6)	
C1	0.7004 (4)	0.2060 (4)	0.53373 (7)	0.0361 (9)	
C2	0.6581 (4)	0.0978 (4)	0.53413 (8)	0.0418 (10)	
H2A	0.579905	0.085380	0.533196	0.050*	
C3	0.7300 (4)	0.0097 (4)	0.53589 (10)	0.0496 (11)	
H3A	0.701999	−0.064416	0.536442	0.060*	
C4	0.8463 (4)	0.0293 (4)	0.53688 (11)	0.0537 (12)	
C5	0.9218 (6)	−0.0627 (6)	0.53717 (18)	0.089 (2)	
H5A	0.893658	−0.136769	0.536834	0.107*	
C6	1.0334 (6)	−0.0449 (6)	0.5379 (2)	0.113 (3)	
H6A	1.083186	−0.106547	0.538366	0.136*	
C7	1.0754 (5)	0.0636 (6)	0.53811 (19)	0.092 (3)	
H7A	1.153776	0.074929	0.539014	0.111*	
C8	1.0064 (4)	0.1537 (5)	0.53701 (12)	0.0591 (14)	
H8A	1.037188	0.226617	0.536235	0.071*	
C9	0.8892 (4)	0.1397 (4)	0.53701 (9)	0.0456 (11)	
C10	0.8124 (4)	0.2319 (4)	0.53615 (8)	0.0373 (9)	
C11	0.8094 (3)	0.4228 (4)	0.51800 (8)	0.0379 (9)	
C12	0.8427 (4)	0.5346 (4)	0.51600 (9)	0.0465 (11)	
H12A	0.813430	0.580538	0.502853	0.056*	
C13	0.9167 (4)	0.5764 (4)	0.53291 (10)	0.0492 (11)	
H13A	0.942837	0.650745	0.530978	0.059*	
C14	0.9554 (4)	0.5108 (4)	0.55342 (9)	0.0474 (11)	
C15	1.0300 (4)	0.5549 (5)	0.57174 (10)	0.0575 (14)	
H15A	1.055261	0.629720	0.570101	0.069*	
C16	1.0661 (4)	0.4915 (6)	0.59175 (10)	0.0643 (16)	
H16A	1.117585	0.521650	0.603679	0.077*	
C17	1.0269 (4)	0.3816 (6)	0.59468 (9)	0.0601 (15)	
H17A	1.050230	0.338638	0.608946	0.072*	
C18	0.9563 (4)	0.3363 (5)	0.57739 (9)	0.0508 (12)	
H18A	0.930467	0.262106	0.579789	0.061*	
C19	0.9200 (4)	0.3976 (4)	0.55571 (8)	0.0426 (10)	
C20	0.8477 (4)	0.3513 (4)	0.53680 (8)	0.0371 (9)	
C21	0.3784 (3)	0.3994 (3)	0.41950 (7)	0.0335 (8)	
C22	0.4624 (4)	0.3994 (4)	0.40081 (7)	0.0362 (9)	
H22A	0.531106	0.436819	0.403744	0.043*	
C23	0.4439 (4)	0.3446 (4)	0.37837 (7)	0.0389 (10)	
H23A	0.499050	0.346153	0.365362	0.047*	
C24	0.3424 (4)	0.2853 (4)	0.37431 (7)	0.0363 (9)	
C25	0.3247 (4)	0.2241 (4)	0.35164 (8)	0.0417 (10)	
H25A	0.380036	0.224733	0.338670	0.050*	
C26	0.2295 (5)	0.1645 (4)	0.34819 (8)	0.0500 (12)	
H26A	0.217885	0.125179	0.332686	0.060*	
C27	0.1484 (4)	0.1608 (4)	0.36740 (9)	0.0481 (11)	
H27A	0.083146	0.116987	0.365020	0.058*	
C28	0.1619 (4)	0.2196 (4)	0.38956 (8)	0.0427 (10)	
H28A	0.106033	0.216276	0.402372	0.051*	
C29	0.2589 (4)	0.2855 (4)	0.39357 (7)	0.0355 (9)	
C30	0.2764 (4)	0.3487 (4)	0.41655 (7)	0.0333 (9)	
C31	0.2185 (4)	0.3375 (4)	0.46197 (7)	0.0339 (9)	
C32	0.1400 (4)	0.3397 (4)	0.48183 (7)	0.0373 (9)	
H32A	0.162733	0.324610	0.498837	0.045*	
C33	0.0315 (4)	0.3634 (4)	0.47672 (8)	0.0393 (9)	
H33A	−0.022239	0.360296	0.490023	0.047*	
C34	−0.0025 (4)	0.3928 (4)	0.45168 (8)	0.0388 (9)	
C35	−0.1140 (4)	0.4256 (4)	0.44645 (9)	0.0450 (11)	
H35A	−0.167964	0.422774	0.459712	0.054*	
C36	−0.1453 (4)	0.4611 (5)	0.42290 (9)	0.0551 (13)	
H36A	−0.220192	0.482903	0.419637	0.066*	
C37	−0.0645 (4)	0.4647 (5)	0.40342 (9)	0.0516 (12)	
H37A	−0.085610	0.489889	0.386940	0.062*	
C38	0.0438 (4)	0.4330 (4)	0.40761 (8)	0.0427 (10)	
H38A	0.096525	0.437562	0.394122	0.051*	
C39	0.0780 (4)	0.3936 (4)	0.43172 (8)	0.0361 (9)	
C40	0.1903 (3)	0.3591 (3)	0.43689 (7)	0.0323 (8)	
C41	0.5918 (4)	0.2427 (4)	0.48152 (8)	0.0401 (10)	
H41A	0.664247	0.207405	0.477382	0.048*	
H41B	0.541652	0.184854	0.488634	0.048*	
C42	0.5401 (4)	0.2936 (4)	0.45746 (7)	0.0373 (9)	
H42A	0.507843	0.234114	0.446639	0.045*	
H42B	0.597990	0.333187	0.447484	0.045*	
Cl2	−0.2595 (4)	0.2896 (5)	0.33679 (12)	0.169 (2)	0.700 (6)
Cl3	−0.1596 (4)	0.1350 (3)	0.36972 (10)	0.1311 (17)	0.700 (6)
C1S	−0.1387 (12)	0.2258 (12)	0.3414 (3)	0.097 (4)	0.700 (6)
H1SA	−0.078690	0.280818	0.344595	0.116*	0.700 (6)
H1SB	−0.118299	0.180567	0.326295	0.116*	0.700 (6)
Cl4	−0.1512 (15)	0.0882 (16)	0.4023 (3)	0.218 (7)	0.300 (6)
Cl5	−0.1366 (19)	0.188 (2)	0.3518 (3)	0.222 (7)	0.300 (6)
C2S	−0.182 (3)	0.200 (2)	0.3855 (4)	0.125 (7)	0.300 (6)
H2SB	−0.144838	0.265583	0.393264	0.150*	0.300 (6)
H2SA	−0.263197	0.212535	0.386018	0.150*	0.300 (6)

### Atomic displacement parameters (Å^2^)

**Table:**

	*U*^11^	*U*^22^	*U*^33^	*U*^12^	*U*^13^	*U*^23^
Fe1	0.0364 (3)	0.0364 (3)	0.0193 (4)	0.0046 (3)	−0.0013 (2)	0.0013 (2)
Cl1	0.0458 (5)	0.0441 (5)	0.0298 (5)	0.0011 (4)	−0.0063 (4)	−0.0011 (4)
P1	0.0381 (5)	0.0413 (5)	0.0218 (5)	0.0066 (4)	−0.0013 (4)	0.0015 (4)
P2	0.0380 (5)	0.0394 (5)	0.0197 (4)	0.0048 (4)	−0.0016 (4)	0.0007 (4)
O1	0.0394 (15)	0.0482 (16)	0.0240 (12)	0.0076 (12)	0.0029 (12)	0.0035 (13)
O2	0.0358 (15)	0.0446 (16)	0.0255 (13)	0.0077 (13)	0.0001 (11)	0.0056 (12)
O3	0.0424 (15)	0.0400 (15)	0.0192 (12)	0.0026 (12)	−0.0036 (11)	0.0007 (10)
O4	0.0382 (15)	0.0406 (15)	0.0244 (12)	0.0027 (12)	−0.0025 (11)	0.0038 (11)
C1	0.037 (2)	0.046 (2)	0.0252 (18)	0.0081 (19)	0.0008 (16)	0.0047 (17)
C2	0.042 (2)	0.049 (3)	0.034 (2)	0.000 (2)	−0.0020 (19)	0.0056 (19)
C3	0.052 (3)	0.042 (3)	0.054 (3)	0.003 (2)	0.002 (2)	0.013 (2)
C4	0.048 (3)	0.047 (3)	0.066 (3)	0.014 (2)	0.010 (2)	0.015 (2)
C5	0.064 (4)	0.050 (3)	0.153 (7)	0.020 (3)	0.022 (4)	0.029 (4)
C6	0.053 (4)	0.065 (4)	0.221 (10)	0.025 (3)	0.039 (5)	0.048 (5)
C7	0.042 (3)	0.066 (4)	0.169 (8)	0.016 (3)	0.021 (4)	0.039 (4)
C8	0.039 (3)	0.057 (3)	0.081 (4)	0.005 (2)	0.010 (2)	0.020 (3)
C9	0.038 (2)	0.052 (3)	0.047 (2)	0.006 (2)	0.0070 (19)	0.015 (2)
C10	0.038 (2)	0.045 (2)	0.0290 (19)	0.0054 (18)	0.0019 (16)	0.0065 (17)
C11	0.033 (2)	0.049 (3)	0.031 (2)	0.0058 (19)	0.0021 (17)	0.0019 (18)
C12	0.046 (2)	0.046 (3)	0.047 (3)	0.007 (2)	0.004 (2)	0.008 (2)
C13	0.045 (3)	0.045 (3)	0.058 (3)	0.003 (2)	0.001 (2)	−0.006 (2)
C14	0.040 (2)	0.055 (3)	0.047 (3)	0.005 (2)	0.004 (2)	−0.006 (2)
C15	0.043 (3)	0.073 (4)	0.057 (3)	0.000 (3)	−0.001 (2)	−0.022 (3)
C16	0.040 (2)	0.109 (5)	0.044 (3)	0.003 (3)	−0.005 (2)	−0.018 (3)
C17	0.039 (3)	0.104 (5)	0.037 (2)	0.012 (3)	−0.002 (2)	0.001 (3)
C18	0.040 (2)	0.078 (4)	0.034 (2)	0.005 (2)	−0.0028 (19)	0.005 (2)
C19	0.033 (2)	0.060 (3)	0.036 (2)	0.003 (2)	0.0031 (17)	−0.001 (2)
C20	0.033 (2)	0.048 (2)	0.030 (2)	0.0043 (19)	0.0025 (16)	0.0026 (18)
C21	0.042 (2)	0.038 (2)	0.0201 (17)	0.0032 (17)	−0.0021 (15)	−0.0026 (15)
C22	0.039 (2)	0.044 (2)	0.0254 (18)	−0.0003 (18)	−0.0006 (16)	0.0036 (16)
C23	0.046 (2)	0.050 (2)	0.0207 (18)	0.006 (2)	0.0026 (16)	0.0035 (17)
C24	0.046 (2)	0.041 (2)	0.0213 (18)	0.0038 (19)	0.0014 (16)	0.0027 (16)
C25	0.057 (3)	0.045 (2)	0.0236 (19)	0.002 (2)	0.0025 (18)	−0.0011 (17)
C26	0.073 (3)	0.051 (3)	0.026 (2)	−0.001 (2)	−0.007 (2)	−0.0067 (19)
C27	0.056 (3)	0.051 (3)	0.037 (2)	−0.010 (2)	−0.009 (2)	−0.003 (2)
C28	0.046 (2)	0.053 (3)	0.029 (2)	−0.003 (2)	0.0014 (18)	−0.0013 (18)
C29	0.043 (2)	0.040 (2)	0.0235 (18)	0.0018 (18)	−0.0021 (16)	0.0001 (16)
C30	0.041 (2)	0.037 (2)	0.0221 (17)	0.0039 (18)	−0.0010 (16)	0.0013 (16)
C31	0.038 (2)	0.037 (2)	0.0266 (19)	0.0005 (17)	−0.0017 (16)	0.0000 (16)
C32	0.042 (2)	0.046 (2)	0.0237 (18)	0.0016 (19)	−0.0004 (16)	0.0028 (17)
C33	0.043 (2)	0.046 (2)	0.029 (2)	0.002 (2)	0.0062 (17)	0.0038 (17)
C34	0.041 (2)	0.043 (2)	0.033 (2)	0.0012 (19)	0.0018 (18)	0.0005 (17)
C35	0.037 (2)	0.058 (3)	0.040 (2)	0.004 (2)	0.0042 (19)	0.004 (2)
C36	0.041 (2)	0.080 (4)	0.044 (3)	0.012 (3)	0.000 (2)	0.013 (3)
C37	0.045 (2)	0.076 (4)	0.033 (2)	0.008 (3)	−0.0032 (19)	0.014 (2)
C38	0.043 (2)	0.058 (3)	0.028 (2)	0.003 (2)	−0.0006 (17)	0.0039 (19)
C39	0.041 (2)	0.041 (2)	0.0261 (19)	−0.0013 (19)	0.0011 (16)	−0.0008 (17)
C40	0.036 (2)	0.035 (2)	0.0253 (18)	0.0004 (17)	−0.0027 (16)	−0.0011 (15)
C41	0.049 (3)	0.042 (2)	0.029 (2)	0.009 (2)	0.0003 (18)	0.0005 (17)
C42	0.043 (2)	0.045 (2)	0.0238 (18)	0.0063 (19)	−0.0004 (16)	−0.0047 (17)
Cl2	0.100 (3)	0.182 (5)	0.225 (5)	0.005 (3)	−0.006 (3)	0.093 (4)
Cl3	0.100 (2)	0.102 (3)	0.191 (5)	−0.0034 (19)	−0.034 (3)	0.026 (3)
C1S	0.098 (7)	0.088 (7)	0.105 (8)	−0.010 (6)	−0.023 (6)	0.051 (6)
Cl4	0.190 (11)	0.248 (14)	0.216 (13)	−0.008 (12)	0.029 (11)	0.023 (11)
Cl5	0.201 (12)	0.225 (14)	0.241 (15)	0.000 (12)	−0.008 (13)	−0.052 (12)
C2S	0.135 (14)	0.110 (14)	0.130 (14)	−0.003 (13)	0.025 (13)	−0.071 (12)

### Geometric parameters (Å, º)

**Table:**

Fe1—P2	2.1594 (11)	C19—C20	1.429 (6)
Fe1—P2^i^	2.1595 (11)	C21—C30	1.375 (6)
Fe1—P1^i^	2.1952 (10)	C21—C22	1.405 (6)
Fe1—P1	2.1952 (10)	C22—C23	1.366 (6)
Fe1—Cl1^i^	2.3422 (11)	C22—H22A	0.9500
Fe1—Cl1	2.3423 (11)	C23—C24	1.426 (7)
P1—O2	1.613 (3)	C23—H23A	0.9500
P1—O1	1.616 (3)	C24—C25	1.413 (6)
P1—C41	1.821 (4)	C24—C29	1.422 (6)
P2—O3	1.611 (3)	C25—C26	1.359 (7)
P2—O4	1.632 (3)	C25—H25A	0.9500
P2—C42	1.821 (4)	C26—C27	1.401 (8)
O1—C11	1.386 (5)	C26—H26A	0.9500
O2—C1	1.401 (5)	C27—C28	1.369 (6)
O3—C21	1.400 (5)	C27—H27A	0.9500
O4—C31	1.398 (5)	C28—C29	1.420 (6)
C1—C10	1.384 (6)	C28—H28A	0.9500
C1—C2	1.393 (7)	C29—C30	1.439 (6)
C2—C3	1.366 (7)	C30—C40	1.489 (6)
C2—H2A	0.9500	C31—C40	1.383 (6)
C3—C4	1.415 (7)	C31—C32	1.404 (6)
C3—H3A	0.9500	C32—C33	1.358 (7)
C4—C9	1.419 (8)	C32—H32A	0.9500
C4—C5	1.426 (8)	C33—C34	1.419 (6)
C5—C6	1.355 (10)	C33—H33A	0.9500
C5—H5A	0.9500	C34—C35	1.420 (7)
C6—C7	1.395 (11)	C34—C39	1.423 (6)
C6—H6A	0.9500	C35—C36	1.359 (7)
C7—C8	1.361 (8)	C35—H35A	0.9500
C7—H7A	0.9500	C36—C37	1.409 (7)
C8—C9	1.415 (7)	C36—H36A	0.9500
C8—H8A	0.9500	C37—C38	1.370 (7)
C9—C10	1.439 (6)	C37—H37A	0.9500
C10—C20	1.493 (7)	C38—C39	1.411 (6)
C11—C20	1.385 (6)	C38—H38A	0.9500
C11—C12	1.401 (7)	C39—C40	1.434 (6)
C12—C13	1.350 (7)	C41—C42	1.532 (6)
C12—H12A	0.9500	C41—H41A	0.9900
C13—C14	1.411 (7)	C41—H41B	0.9900
C13—H13A	0.9500	C42—H42A	0.9900
C14—C15	1.415 (7)	C42—H42B	0.9900
C14—C19	1.426 (8)	Cl2—C1S	1.656 (15)
C15—C16	1.366 (9)	Cl3—C1S	1.859 (11)
C15—H15A	0.9500	C1S—H1SA	0.9900
C16—C17	1.408 (10)	C1S—H1SB	0.9900
C16—H16A	0.9500	Cl4—C2S	1.644 (18)
C17—C18	1.354 (8)	Cl5—C2S	1.852 (15)
C17—H17A	0.9500	C2S—H2SB	0.9900
C18—C19	1.422 (7)	C2S—H2SA	0.9900
C18—H18A	0.9500		
			
P2—Fe1—P2^i^	108.49 (7)	C11—C20—C19	117.0 (4)
P2—Fe1—P1^i^	93.40 (4)	C11—C20—C10	118.9 (4)
P2^i^—Fe1—P1^i^	85.30 (4)	C19—C20—C10	124.0 (4)
P2—Fe1—P1	85.30 (4)	C30—C21—O3	120.0 (3)
P2^i^—Fe1—P1	93.40 (4)	C30—C21—C22	124.0 (4)
P1^i^—Fe1—P1	177.78 (7)	O3—C21—C22	115.9 (4)
P2—Fe1—Cl1^i^	81.43 (4)	C23—C22—C21	119.0 (4)
P2^i^—Fe1—Cl1^i^	170.01 (5)	C23—C22—H22A	120.5
P1^i^—Fe1—Cl1^i^	93.07 (4)	C21—C22—H22A	120.5
P1—Fe1—Cl1^i^	88.52 (4)	C22—C23—C24	120.5 (4)
P2—Fe1—Cl1	170.02 (5)	C22—C23—H23A	119.8
P2^i^—Fe1—Cl1	81.43 (4)	C24—C23—H23A	119.8
P1^i^—Fe1—Cl1	88.51 (4)	C25—C24—C29	119.6 (4)
P1—Fe1—Cl1	93.07 (4)	C25—C24—C23	120.8 (4)
Cl1^i^—Fe1—Cl1	88.69 (6)	C29—C24—C23	119.6 (4)
O2—P1—O1	100.60 (15)	C26—C25—C24	120.7 (4)
O2—P1—C41	104.87 (18)	C26—C25—H25A	119.6
O1—P1—C41	98.46 (19)	C24—C25—H25A	119.6
O2—P1—Fe1	118.67 (11)	C25—C26—C27	120.2 (4)
O1—P1—Fe1	120.18 (12)	C25—C26—H26A	119.9
C41—P1—Fe1	111.26 (15)	C27—C26—H26A	119.9
O3—P2—O4	100.52 (14)	C28—C27—C26	120.8 (4)
O3—P2—C42	104.53 (17)	C28—C27—H27A	119.6
O4—P2—C42	98.48 (18)	C26—C27—H27A	119.6
O3—P2—Fe1	117.25 (11)	C27—C28—C29	120.6 (4)
O4—P2—Fe1	122.98 (11)	C27—C28—H28A	119.7
C42—P2—Fe1	110.23 (14)	C29—C28—H28A	119.7
C11—O1—P1	120.1 (2)	C28—C29—C24	118.0 (4)
C1—O2—P1	117.1 (2)	C28—C29—C30	122.4 (4)
C21—O3—P2	118.2 (2)	C24—C29—C30	119.6 (4)
C31—O4—P2	121.4 (3)	C21—C30—C29	117.2 (4)
C10—C1—C2	124.1 (4)	C21—C30—C40	119.9 (4)
C10—C1—O2	119.6 (4)	C29—C30—C40	122.9 (4)
C2—C1—O2	116.2 (4)	C40—C31—O4	120.1 (4)
C3—C2—C1	119.4 (4)	C40—C31—C32	122.6 (4)
C3—C2—H2A	120.3	O4—C31—C32	117.2 (3)
C1—C2—H2A	120.3	C33—C32—C31	120.0 (4)
C2—C3—C4	119.7 (5)	C33—C32—H32A	120.0
C2—C3—H3A	120.1	C31—C32—H32A	120.0
C4—C3—H3A	120.1	C32—C33—C34	120.6 (4)
C3—C4—C9	120.8 (4)	C32—C33—H33A	119.7
C3—C4—C5	119.8 (5)	C34—C33—H33A	119.7
C9—C4—C5	119.4 (5)	C33—C34—C35	121.1 (4)
C6—C5—C4	120.3 (7)	C33—C34—C39	119.2 (4)
C6—C5—H5A	119.8	C35—C34—C39	119.6 (4)
C4—C5—H5A	119.8	C36—C35—C34	121.5 (4)
C5—C6—C7	120.2 (6)	C36—C35—H35A	119.3
C5—C6—H6A	119.9	C34—C35—H35A	119.3
C7—C6—H6A	119.9	C35—C36—C37	118.6 (5)
C8—C7—C6	121.3 (6)	C35—C36—H36A	120.7
C8—C7—H7A	119.3	C37—C36—H36A	120.7
C6—C7—H7A	119.3	C38—C37—C36	121.8 (4)
C7—C8—C9	120.6 (5)	C38—C37—H37A	119.1
C7—C8—H8A	119.7	C36—C37—H37A	119.1
C9—C8—H8A	119.7	C37—C38—C39	120.8 (4)
C8—C9—C4	118.0 (4)	C37—C38—H38A	119.6
C8—C9—C10	122.9 (5)	C39—C38—H38A	119.6
C4—C9—C10	119.0 (4)	C38—C39—C34	117.7 (4)
C1—C10—C9	116.8 (4)	C38—C39—C40	122.6 (4)
C1—C10—C20	119.4 (4)	C34—C39—C40	119.7 (4)
C9—C10—C20	123.7 (4)	C31—C40—C39	117.6 (4)
C20—C11—O1	119.0 (4)	C31—C40—C30	119.8 (4)
C20—C11—C12	123.4 (4)	C39—C40—C30	122.6 (3)
O1—C11—C12	117.4 (4)	C42—C41—P1	107.9 (3)
C13—C12—C11	119.5 (5)	C42—C41—H41A	110.1
C13—C12—H12A	120.2	P1—C41—H41A	110.1
C11—C12—H12A	120.2	C42—C41—H41B	110.1
C12—C13—C14	120.6 (5)	P1—C41—H41B	110.1
C12—C13—H13A	119.7	H41A—C41—H41B	108.4
C14—C13—H13A	119.7	C41—C42—P2	108.2 (3)
C13—C14—C15	121.1 (5)	C41—C42—H42A	110.1
C13—C14—C19	119.7 (4)	P2—C42—H42A	110.1
C15—C14—C19	119.1 (5)	C41—C42—H42B	110.1
C16—C15—C14	120.9 (6)	P2—C42—H42B	110.1
C16—C15—H15A	119.5	H42A—C42—H42B	108.4
C14—C15—H15A	119.5	Cl2—C1S—Cl3	105.6 (8)
C15—C16—C17	119.9 (5)	Cl2—C1S—H1SA	110.6
C15—C16—H16A	120.0	Cl3—C1S—H1SA	110.6
C17—C16—H16A	120.0	Cl2—C1S—H1SB	110.6
C18—C17—C16	120.7 (5)	Cl3—C1S—H1SB	110.6
C18—C17—H17A	119.7	H1SA—C1S—H1SB	108.7
C16—C17—H17A	119.7	Cl4—C2S—Cl5	112.5 (14)
C17—C18—C19	121.3 (6)	Cl4—C2S—H2SB	109.1
C17—C18—H18A	119.3	Cl5—C2S—H2SB	109.1
C19—C18—H18A	119.3	Cl4—C2S—H2SA	109.1
C18—C19—C14	117.9 (5)	Cl5—C2S—H2SA	109.1
C18—C19—C20	122.7 (5)	H2SB—C2S—H2SA	107.8
C14—C19—C20	119.4 (4)		
			
O2—P1—O1—C11	−44.5 (3)	C1—C10—C20—C19	130.0 (4)
C41—P1—O1—C11	−151.5 (3)	C9—C10—C20—C19	−52.3 (6)
Fe1—P1—O1—C11	87.8 (3)	P2—O3—C21—C30	76.9 (4)
O1—P1—O2—C1	−49.0 (3)	P2—O3—C21—C22	−105.7 (4)
C41—P1—O2—C1	52.8 (3)	C30—C21—C22—C23	−1.0 (7)
Fe1—P1—O2—C1	177.7 (3)	O3—C21—C22—C23	−178.3 (4)
O4—P2—O3—C21	−50.9 (3)	C21—C22—C23—C24	−2.1 (6)
C42—P2—O3—C21	50.8 (3)	C22—C23—C24—C25	−177.0 (4)
Fe1—P2—O3—C21	173.2 (2)	C22—C23—C24—C29	1.5 (6)
O3—P2—O4—C31	−40.3 (3)	C29—C24—C25—C26	−0.8 (7)
C42—P2—O4—C31	−146.9 (3)	C23—C24—C25—C26	177.7 (4)
Fe1—P2—O4—C31	92.2 (3)	C24—C25—C26—C27	−1.5 (8)
P1—O2—C1—C10	76.8 (4)	C25—C26—C27—C28	2.0 (8)
P1—O2—C1—C2	−105.8 (4)	C26—C27—C28—C29	−0.1 (8)
C10—C1—C2—C3	−3.0 (7)	C27—C28—C29—C24	−2.1 (7)
O2—C1—C2—C3	179.7 (4)	C27—C28—C29—C30	179.9 (4)
C1—C2—C3—C4	−1.0 (7)	C25—C24—C29—C28	2.6 (6)
C2—C3—C4—C9	2.3 (8)	C23—C24—C29—C28	−176.0 (4)
C2—C3—C4—C5	−176.3 (6)	C25—C24—C29—C30	−179.3 (4)
C3—C4—C5—C6	179.5 (8)	C23—C24—C29—C30	2.1 (6)
C9—C4—C5—C6	0.9 (12)	O3—C21—C30—C29	−178.2 (4)
C4—C5—C6—C7	−0.8 (16)	C22—C21—C30—C29	4.6 (6)
C5—C6—C7—C8	−1.3 (16)	O3—C21—C30—C40	0.7 (6)
C6—C7—C8—C9	3.4 (13)	C22—C21—C30—C40	−176.4 (4)
C7—C8—C9—C4	−3.2 (9)	C28—C29—C30—C21	173.0 (4)
C7—C8—C9—C10	179.3 (6)	C24—C29—C30—C21	−5.0 (6)
C3—C4—C9—C8	−177.6 (5)	C28—C29—C30—C40	−5.9 (7)
C5—C4—C9—C8	1.0 (8)	C24—C29—C30—C40	176.1 (4)
C3—C4—C9—C10	0.0 (7)	P2—O4—C31—C40	72.6 (5)
C5—C4—C9—C10	178.7 (6)	P2—O4—C31—C32	−112.1 (4)
C2—C1—C10—C9	5.3 (6)	C40—C31—C32—C33	0.2 (7)
O2—C1—C10—C9	−177.5 (4)	O4—C31—C32—C33	−174.9 (4)
C2—C1—C10—C20	−176.8 (4)	C31—C32—C33—C34	−3.9 (7)
O2—C1—C10—C20	0.4 (6)	C32—C33—C34—C35	−175.4 (5)
C8—C9—C10—C1	173.8 (5)	C32—C33—C34—C39	2.1 (7)
C4—C9—C10—C1	−3.6 (6)	C33—C34—C35—C36	175.5 (5)
C8—C9—C10—C20	−4.0 (7)	C39—C34—C35—C36	−1.9 (8)
C4—C9—C10—C20	178.5 (4)	C34—C35—C36—C37	0.1 (9)
P1—O1—C11—C20	76.4 (4)	C35—C36—C37—C38	0.4 (9)
P1—O1—C11—C12	−108.5 (4)	C36—C37—C38—C39	1.0 (9)
C20—C11—C12—C13	0.7 (7)	C37—C38—C39—C34	−2.8 (7)
O1—C11—C12—C13	−174.1 (4)	C37—C38—C39—C40	179.8 (5)
C11—C12—C13—C14	−3.9 (7)	C33—C34—C39—C38	−174.3 (4)
C12—C13—C14—C15	−177.8 (5)	C35—C34—C39—C38	3.2 (7)
C12—C13—C14—C19	2.7 (7)	C33—C34—C39—C40	3.3 (7)
C13—C14—C15—C16	179.1 (5)	C35—C34—C39—C40	−179.2 (4)
C19—C14—C15—C16	−1.4 (7)	O4—C31—C40—C39	−179.9 (4)
C14—C15—C16—C17	−1.6 (8)	C32—C31—C40—C39	5.1 (6)
C15—C16—C17—C18	2.1 (8)	O4—C31—C40—C30	−1.4 (6)
C16—C17—C18—C19	0.4 (8)	C32—C31—C40—C30	−176.4 (4)
C17—C18—C19—C14	−3.3 (7)	C38—C39—C40—C31	170.8 (4)
C17—C18—C19—C20	178.4 (5)	C34—C39—C40—C31	−6.7 (6)
C13—C14—C19—C18	−176.7 (4)	C38—C39—C40—C30	−7.8 (7)
C15—C14—C19—C18	3.8 (6)	C34—C39—C40—C30	174.8 (4)
C13—C14—C19—C20	1.6 (7)	C21—C30—C40—C31	−49.4 (6)
C15—C14—C19—C20	−177.9 (4)	C29—C30—C40—C31	129.5 (4)
O1—C11—C20—C19	178.3 (4)	C21—C30—C40—C39	129.1 (4)
C12—C11—C20—C19	3.5 (6)	C29—C30—C40—C39	−51.9 (6)
O1—C11—C20—C10	−1.3 (6)	O2—P1—C41—C42	159.7 (3)
C12—C11—C20—C10	−176.1 (4)	O1—P1—C41—C42	−96.9 (3)
C18—C19—C20—C11	173.7 (4)	Fe1—P1—C41—C42	30.2 (3)
C14—C19—C20—C11	−4.5 (6)	P1—C41—C42—P2	−43.1 (4)
C18—C19—C20—C10	−6.7 (7)	O3—P2—C42—C41	167.6 (3)
C14—C19—C20—C10	175.1 (4)	O4—P2—C42—C41	−89.1 (3)
C1—C10—C20—C11	−50.5 (6)	Fe1—P2—C42—C41	40.8 (3)
C9—C10—C20—C11	127.3 (4)		

Symmetry code: (i) *y*, *x*, −*z*+1.

### Hydrogen-bond geometry (Å, º)

*Cg*2 and *Cg*3 are the centroids of the C24–C29 and C31–C40 rings, respectively.

**Table:**

*D*—H···*A*	*D*—H	H···*A*	*D*···*A*	*D*—H···*A*
C32—H32*A*···O4^i^	0.95	2.42	3.280 (5)	150
C35—H35*A*···O1^ii^	0.95	2.38	3.293 (5)	162
C7—H7*A*···*Cg*2^iii^	0.95	2.57	3.516 (6)	178
C17—H17*A*···*Cg*3^iii^	0.95	2.59	3.396 (6)	143

Symmetry codes: (i) *y*, *x*, −*z*+1; (ii) *x*−1, *y*, *z*; (iii) *y*+1, *x*, −*z*+1.
